# Supplementary material for: BGN Secreted by Cancer-Associated Fibroblasts Promotes Esophageal Squamous Cell Carcinoma Progression via Activation of TLR4-Mediated Erk and NF-κB Signaling Pathways
Source: Int J Mol Sci. 2025 Dec 13;26(24):12024. doi: 10.3390/ijms262412024 (PMC12733209; doi:10.3390/ijms262412024)
Supplement: Supplementary file 1 [file ijms-26-12024-s001.zip › ijms-3933823-supplementary.pdf]

Supplementary Figure

BGN Secreted by Cancer-Associated Fibroblasts Promotes Esophageal Squamous Cell Carcinoma Progression via Activation of TLR4-Mediated Erk and NF-κB Signaling Pathways

Hiroki Yokoo, Yu-ichiro Koma, Naozane Nomura, Rikuya Torigoe, Masaki Omori, Takashi Nakanishi, Shoji Miyako, Takaaki Nakanishi, Takayuki Kodama, Manabu Shigeoka, Yoshihiro Kakeji and Masafumi Horie

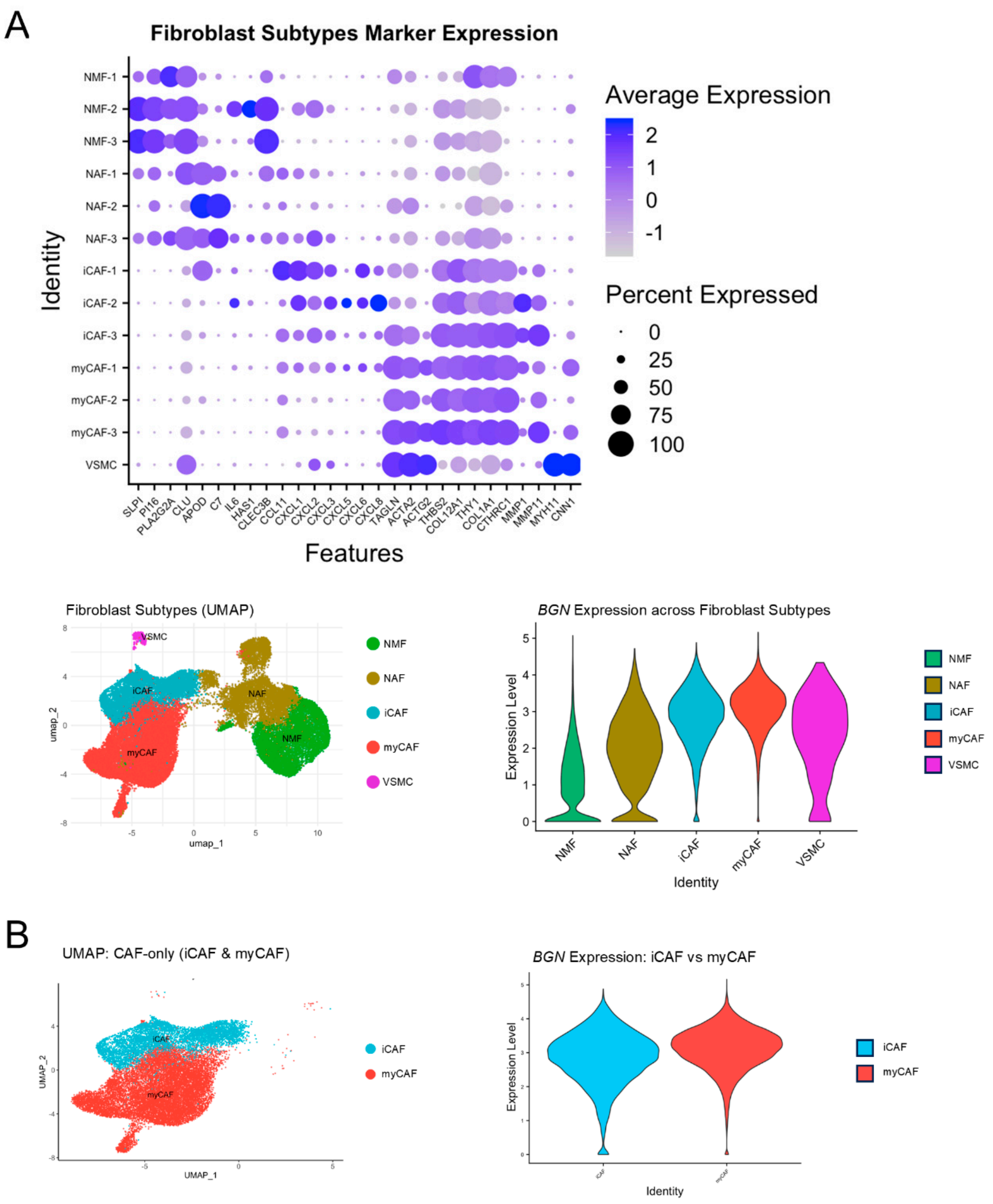

Figure S1. (A) Dot plot showing the expression of marker genes used for fibroblast subtype annotation based on single-cell RNA sequencing (scRNAseq) data. Thirteen fibroblast clusters were identified and classified into five subtypes (myofibroblastic CAFs [myCAF], inflammatory CAFs [iCAF], normal mucosa fibroblasts [NMF], normal activated fibroblasts [NAF], and vascular smooth muscle cells [VSMC]). Uniform Manifold Approximation and Projection (UMAP) visualization showed fibroblast subtypes (myCAF, iCAF, NMF, NAF, and VSMC). Violin plots visualization showed significantly higher *biglycan* (*BGN*) expression in cancer-associated fibroblasts (CAFs) than in normal fibroblasts, with widespread expression across CAF subtypes. (B) UMAP plot restricted to two major subtypes of CAFs (myCAF and iCAF). Violin plot comparing *BGN* expression between myCAF and iCAF, showing higher expression in myCAF (log2 fold change = 0.29, adjusted $p < 0.001$) with expression frequencies of 99.6% and 98.5%, respectively.

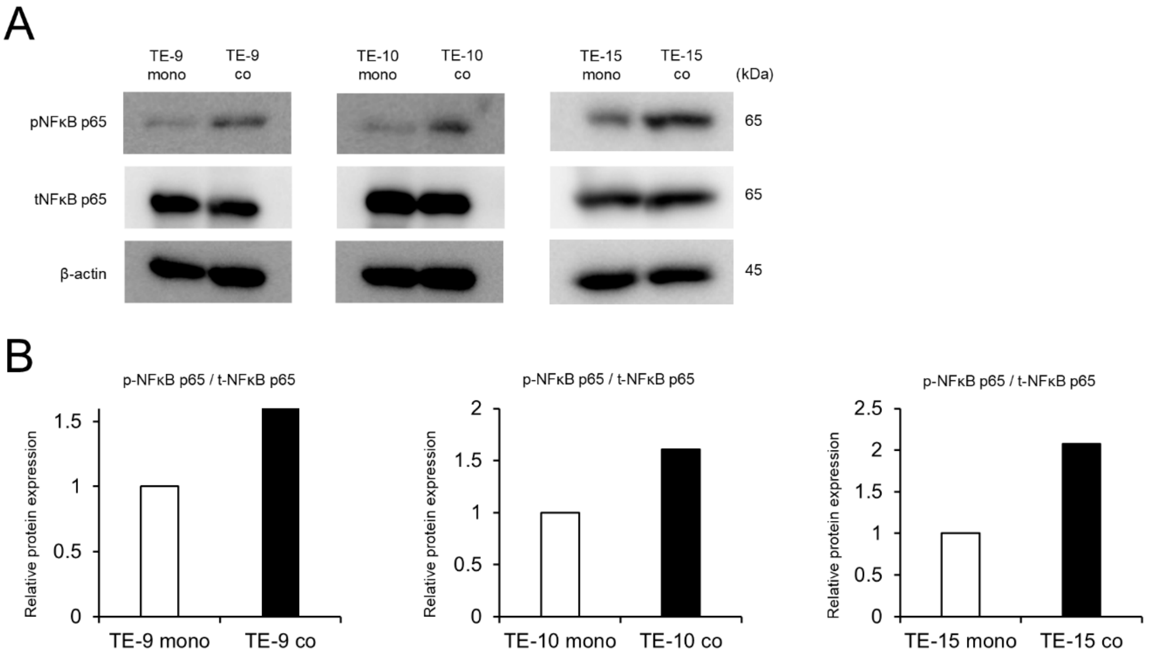

Figure S2. Direct co-culture with mesenchymal stem cells (MSCs) activates the NF-κB pathway in esophageal squamous cell carcinoma (ESCC) cells. (A, B) Protein expression levels of pNF-κB p65 and tNF-κB p65 in TE mono and TE co were analyzed by Western blotting (A), with β-actin as a loading control, and quantified by normalizing pNF-κB p65 to tNF-κB p65 (B).

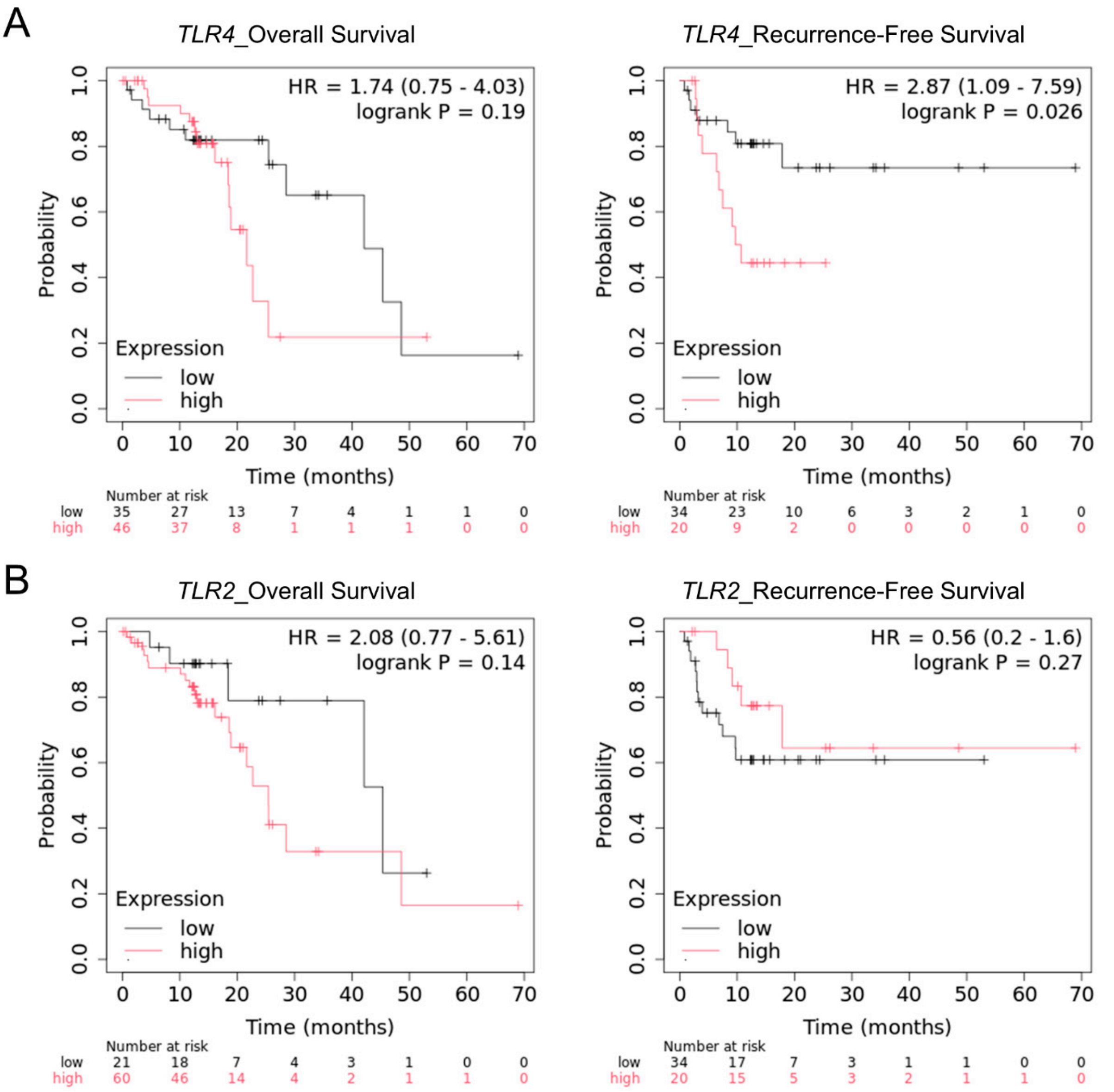

Figure S3 (A, B) Kaplan–Meier survival analysis of public esophageal squamous cell carcinoma (ESCC) data (n = 81, Kaplan–Meier Plotter for *TLR4* (A) and *TLR2* (B)) showed shorter recurrence-free survival in the *TLR4* high-expression group than in the low-expression group (p = 0.026), whereas no significant differences were observed for *TLR2* (p = 0.14 in overall survival and p = 0.27 in recurrence-free survival) and *TLR4* (p = 0.19 in overall survival).

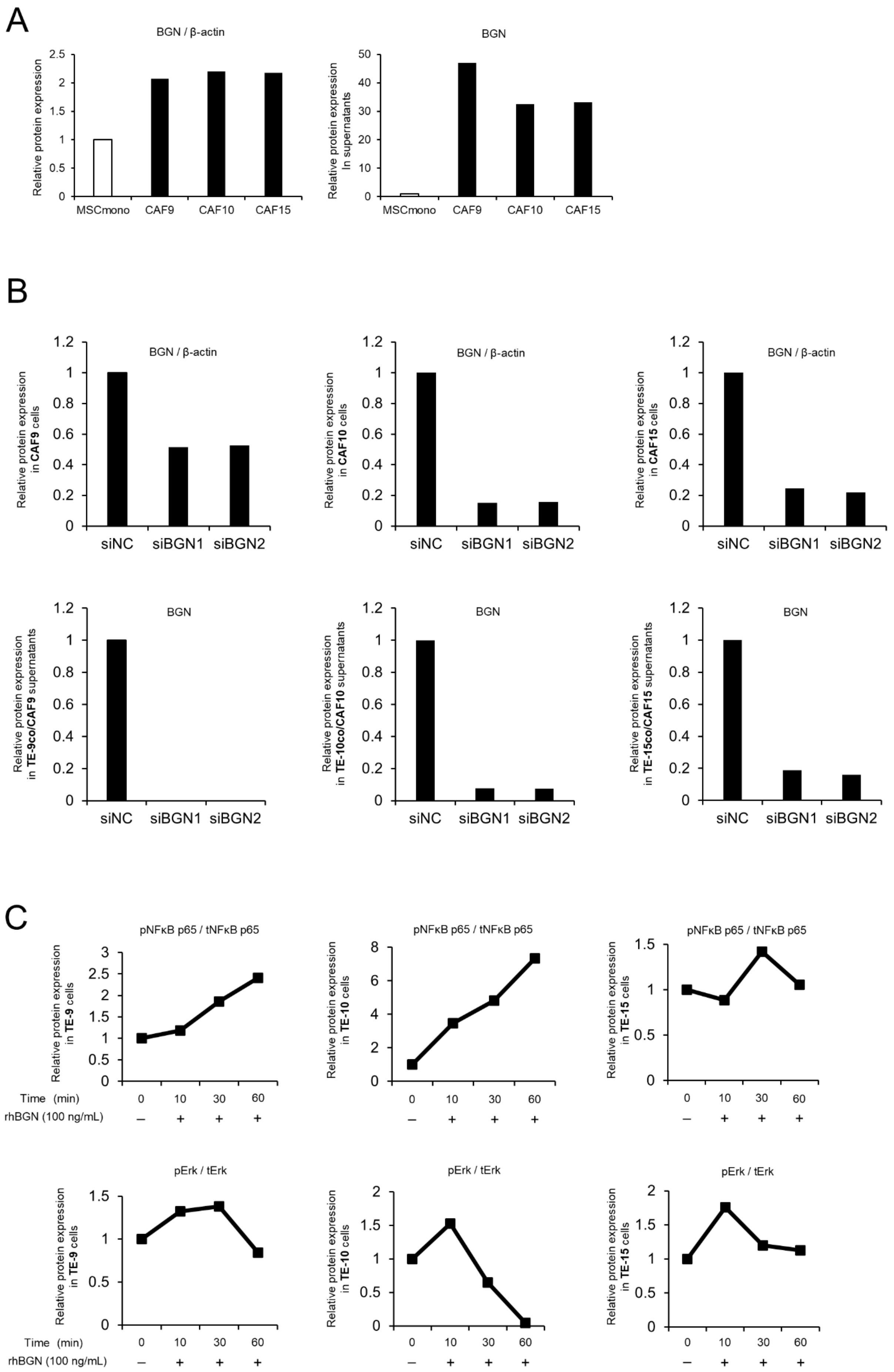
A
BGN / β-actin
Relative protein expression
MSCmono
CAF9
CAF10
CAF15
BGN
Relative protein expression In supernatants
B
BGN / β-actin
Relative protein expression in CAF9 cells
Relative protein expression in CAF10 cells
Relative protein expression in CAF15 cells
siNC
siBGN1
siBGN2
BGN
Relative protein expression in TE-9co/CAF9 supernatants
Relative protein expression in TE-10co/CAF10 supernatants
Relative protein expression in TE-15co/CAF15 supernatants
C
pNFκB p65 / tNFκB p65
Relative protein expression in TE-9 cells
Relative protein expression in TE-10 cells
Relative protein expression in TE-15 cells
Time (min)
rhBGN (100 ng/mL)
pErk / tErk

D

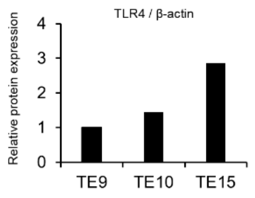

E

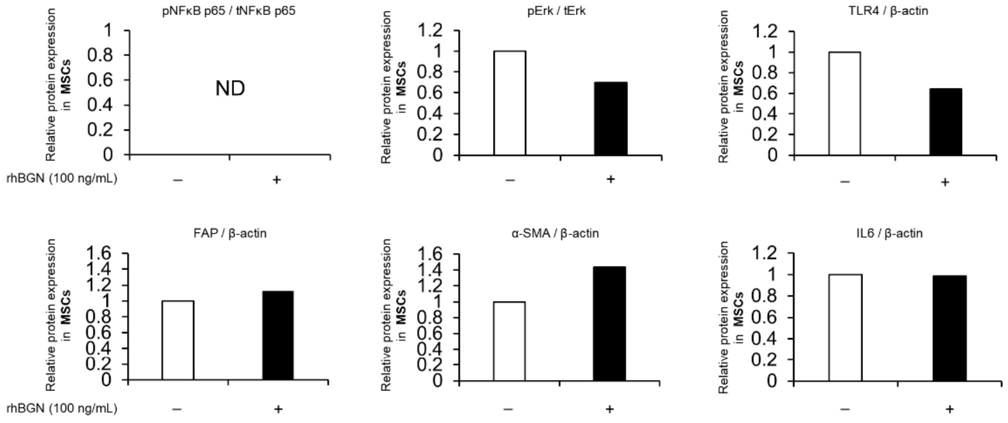

F

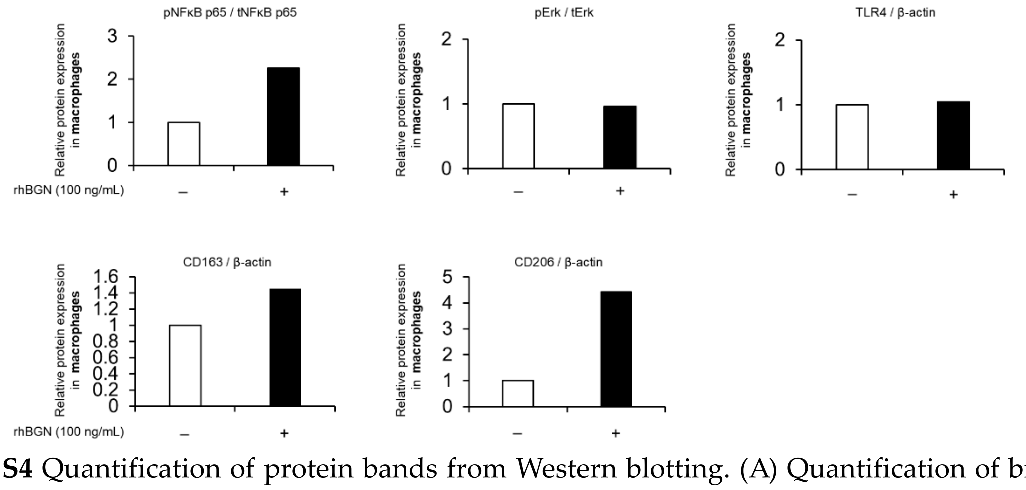

Figure S4 Quantification of protein bands from Western blotting. (A) Quantification of biglycan (BGN) protein levels in cell lysates and supernatants from Figure 1E, F. Lysate protein levels were normalized to β-actin. (B) Quantification of BGN protein levels in cell lysates and supernatants from Figure 2B, C. Lysate protein levels were normalized to β-actin. (C) Quantification of pNF-κB p65 and pErk protein levels from Figure 3A, normalized to their respective total proteins (tNF-κB p65 and tErk). (D) Quantification of TLR4 protein levels from Figure 4A, normalized to β-actin. (E) Quantification of pNF-κB p65, pErk, TLR4, FAP, αSMA, and IL6 protein levels from Figure 5D. pNF-κB p65 and pErk were normalized to their respective total proteins (tNF-κB p65 and tErk), and TLR4, FAP, αSMA, and IL6 were normalized to β-actin. (F) Quantification of pNF-κB p65, pErk, TLR4, CD163, and CD206 protein levels from Figure 5I. pNF-κB p65 and pErk were normalized to their respective total proteins (tNF-κB p65 and tErk), and TLR4, CD163, and CD206 were normalized to β-actin. ND, not detected.

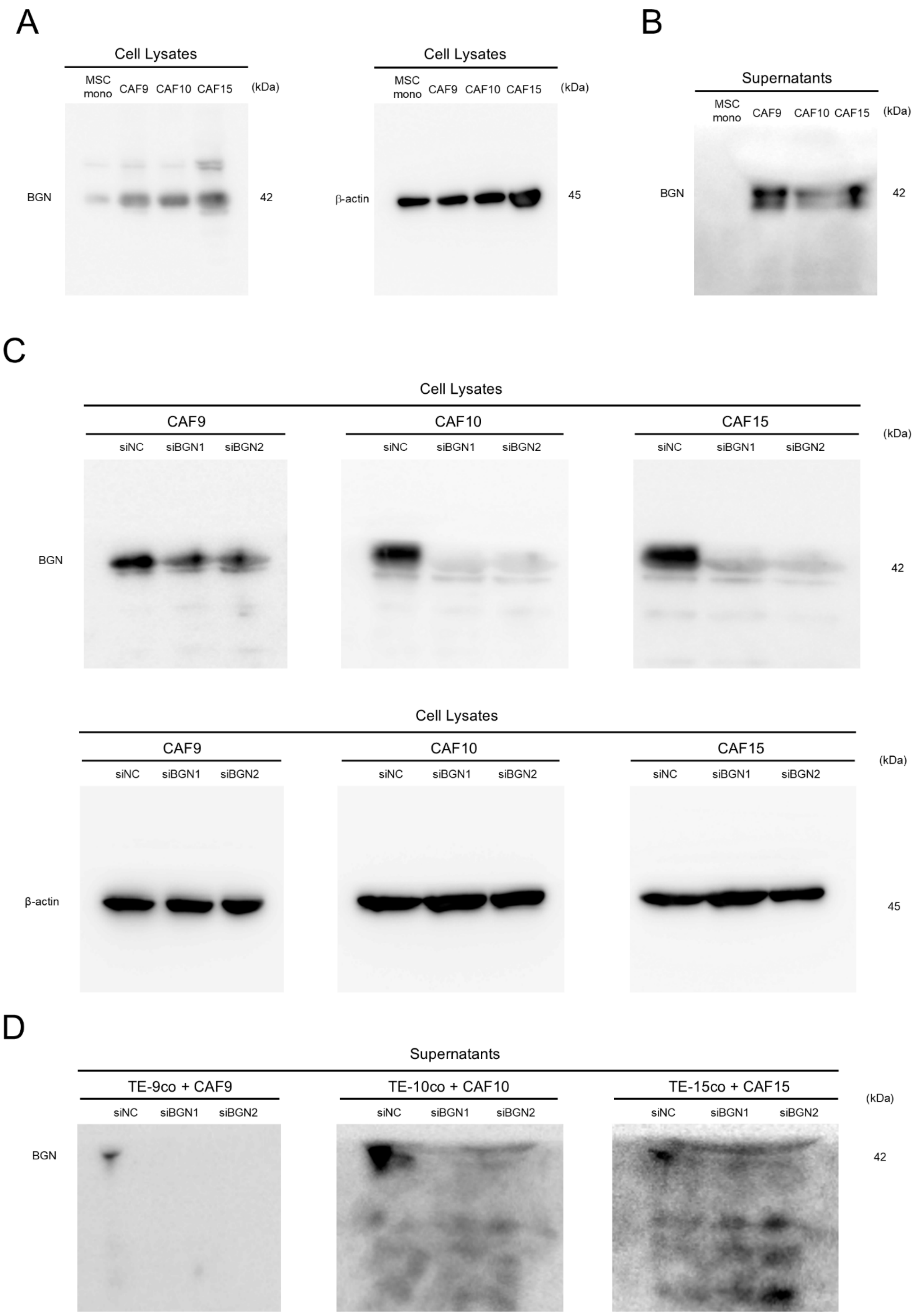
A
Cell Lysates
MSC mono
CAF9
CAF10
CAF15
(kDa)
BGN
42
Cell Lysates
MSC mono
CAF9
CAF10
CAF15
(kDa)
β-actin
45
B
Supernatants
MSC mono
CAF9
CAF10
CAF15
(kDa)
BGN
42
C
Cell Lysates
CAF9
CAF10
CAF15
siNC
siBGN1
siBGN2
(kDa)
BGN
42
Cell Lysates
CAF9
CAF10
CAF15
siNC
siBGN1
siBGN2
(kDa)
β-actin
45
D
Supernatants
TE-9co + CAF9
TE-10co + CAF10
TE-15co + CAF15
siNC
siBGN1
siBGN2
(kDa)
BGN
42

E

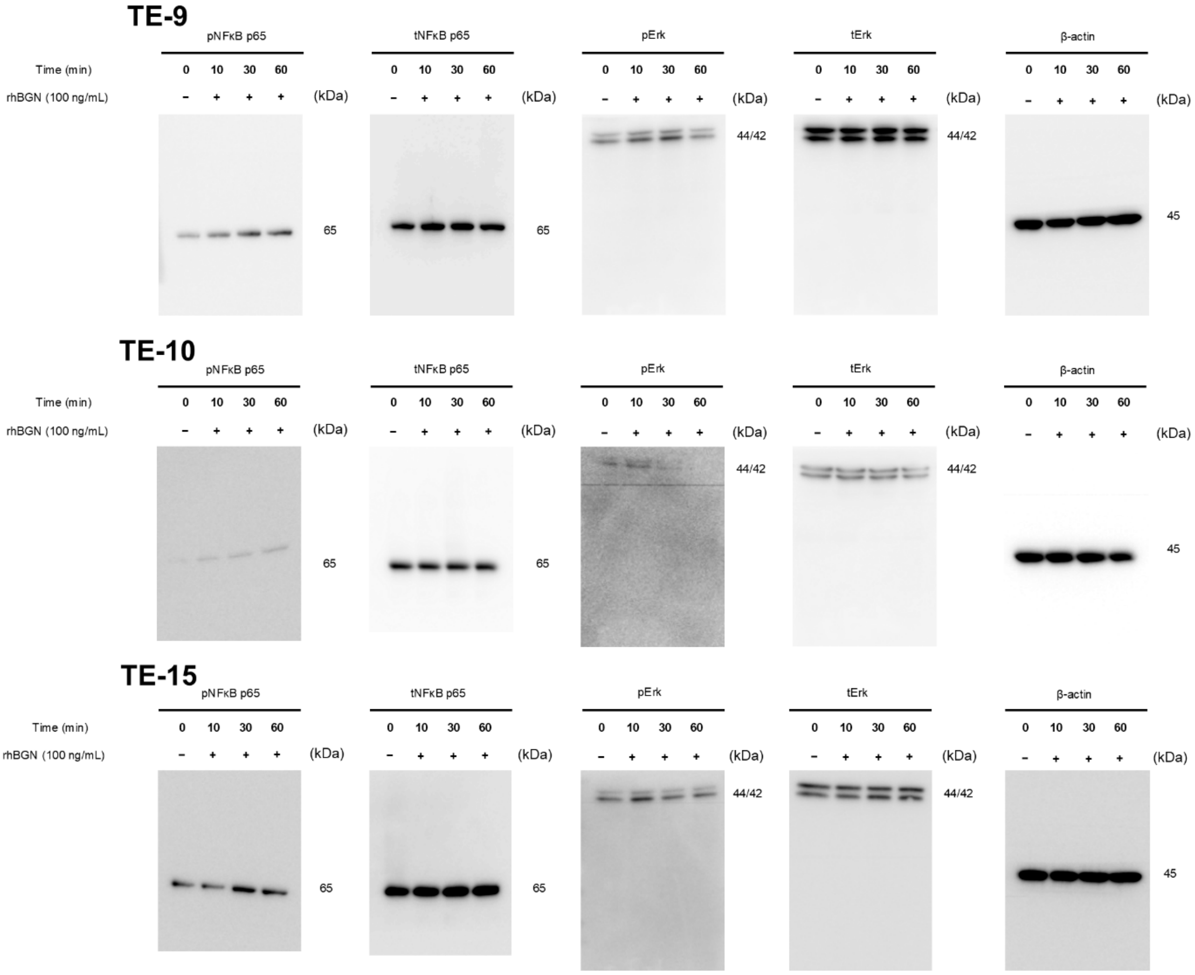

TE-9
pNFκB p65
tNFκB p65
pErk
tErk
β-actin
Time (min)
0 10 30 60
rhBGN (100 ng/mL)
− + + +
(kDa)
65
44/42
45
TE-10
pNFκB p65
tNFκB p65
pErk
tErk
β-actin
Time (min)
0 10 30 60
rhBGN (100 ng/mL)
− + + +
(kDa)
65
44/42
45
TE-15
pNFκB p65
tNFκB p65
pErk
tErk
β-actin
Time (min)
0 10 30 60
rhBGN (100 ng/mL)
− + + +
(kDa)
65
44/42
45

F

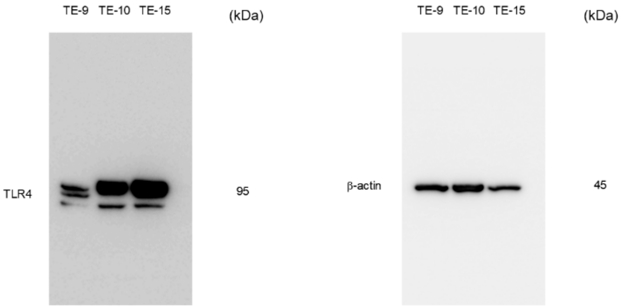

TE-9 TE-10 TE-15
(kDa)
TLR4
95
TE-9 TE-10 TE-15
(kDa)
β-actin
45

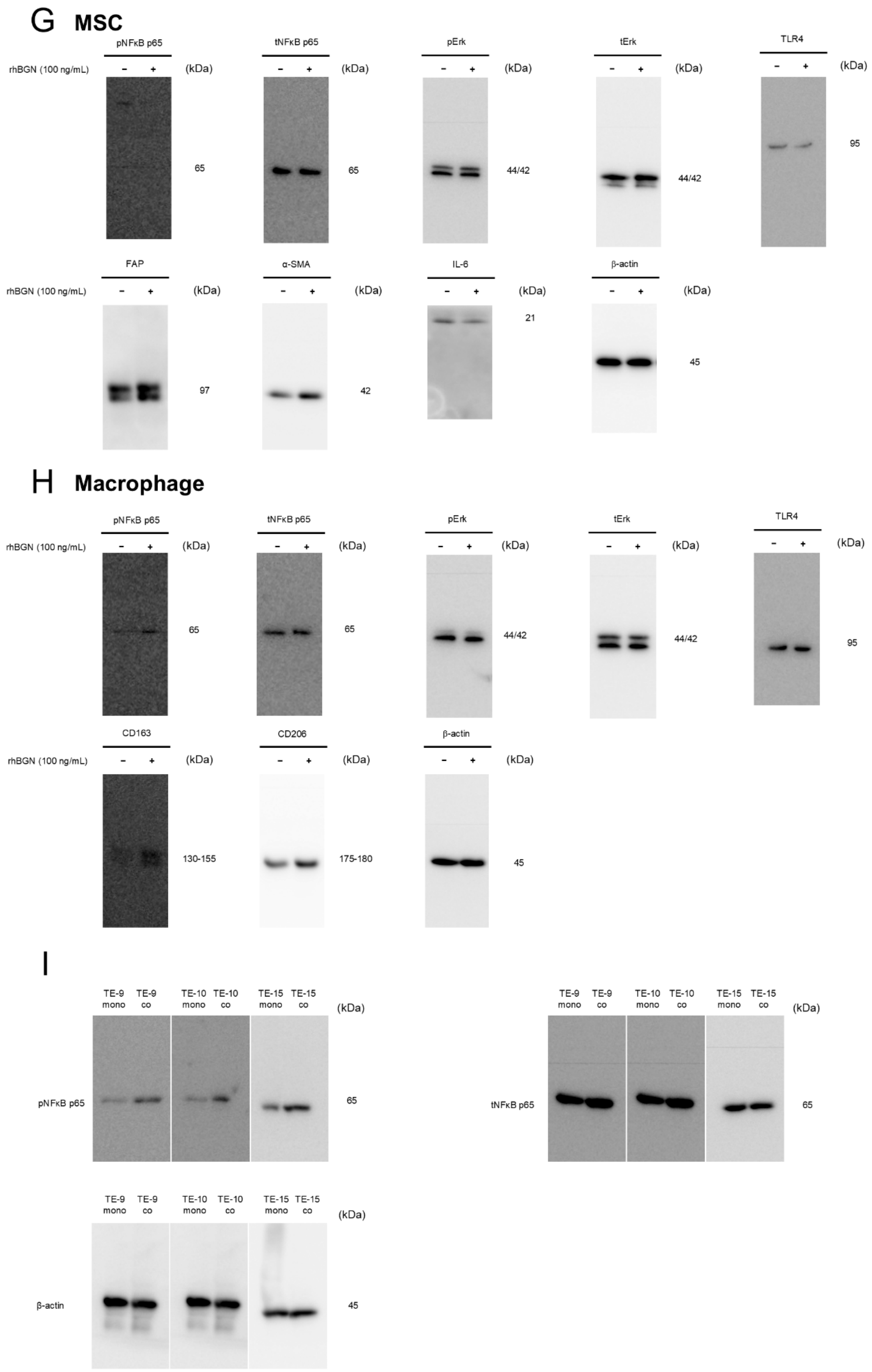

Figure S5 Raw Western blotting images corresponding to Figure 1E, 1F, 2B, 2C, 3A, 4A, 5D, 5I, and S2A are presented as Figure S5A–I, respectively. The protein markers are not depicted on these raw membranes.
